# Outcomes of elective repair of large hiatus hernias in the morbidly obese: a cohort study

**DOI:** 10.1007/s00464-025-11808-z

**Published:** 2025-05-29

**Authors:** Mathew A. Amprayil, Muktar Ahmed, Tanya Irvine, Sarah K. Thompson, Tim Bright, David I. Watson

**Affiliations:** 1 Discipline of Surgery, College of Medicine and Public Health, Flinders University, Adelaide, South Australia Australia; 2 Flinders Health and Medical Research Institute, Flinders University, Adelaide, South Australia Australia; 3 Department of Surgery, Flinders Medical Centre, Bedford Park, Adelaide, South Australia Australia; 4 Flinders Medical Centre, Flinders University Discipline of Surgery, Room 3D211, Bedford Park, Adelaide, South Australia 5042 Australia

**Keywords:** Hiatus hernia, Obese, Surgery, Laparoscopy

## Abstract

**Background:**

Obesity is a risk factor for the development of a large hiatus hernia. Such hernias are often symptomatic and negatively impact quality of life. However, surgeons can be reluctant to operate on obese patients due to concerns of operative complexity, early hernia recurrence, and increased morbidity. To evaluate this, we assessed the perioperative risks and short-term outcomes following surgery in obese and morbidly obese patients.

**Methods:**

Patients who underwent repair of a large hiatus hernia (≥50% intrathoracic stomach) from January 2000 to December 2023 were identified from a prospective database. Patients were categorised based on body mass index (BMI) into 3 groups: non-obese (BMI < 30.0), obese (BMI 30.0–34.9), and morbidly obese (BMI ≥ 35.0). Perioperative and postoperative outcomes were compared.

**Results:**

915 patients were included (non-obese: 519 [56.7%], obese: 276 [30.1%], morbidly obese: 120 [13.1%]). Morbidly obese patients were younger (69.2 vs 65.8 vs 64 years, *p* < 0.001) and more likely to be female (60.9 vs 79.7 vs 83.9%, *p* < 0.001). There were no differences in conversion rates (0.8 vs 0.7 vs 1.7%, *p* = 0.592), operative time (106.4 vs 103.4 vs 113.6 min, *p* = 0.074), or length of stay (2.8 vs 2.48 vs 2.57 days, *p* = 0.063). We found no differences in major complication (4.0 vs 2.9 vs 1.7%, *p* = 0.435) or return to theatre rates (2.7 vs 1.1 vs 1.7%, *p* = 0.475). 90-day mortality rates were low for all groups (0.2 vs 0.4 vs 0%). Postoperative heartburn severity was lowest in non-obese patients (0.94 vs 1.86 vs 1.21, *p* = 0.010). There were no differences in postoperative regurgitation severity (1.02 vs 1.30 vs 1.74, *p* = 0.185) or overall satisfaction (8.74 vs 8.62 vs 8.91, *p* = 0.702).

**Conclusion:**

Large hiatus hernia repair is safe and effective in obese and morbidly obese populations.

**Graphical abstract:**

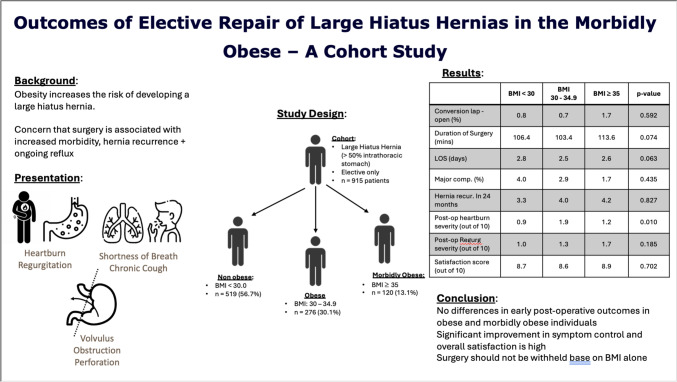

Obesity is a significant risk factor for the development of a large hiatus hernia. [1] In morbidly obese individuals, 37% will have a hiatus hernia of any size and 4.4% have a moderate to large-sized hernia. [2] As the prevalence of obesity has more than doubled worldwide since 1990, the presentation of a large hiatus hernia in obese individuals is becoming more common. [3] In Australia, it is estimated that nearly one in three adults are obese, meaning surgeons are increasingly facing the challenge of operating on larger patients. [4] However, surgeons may be reluctant to offer antireflux surgery to obese patients for several reasons. These concerns include access challenges, increased perioperative morbidity and mortality risks, concern about an increased risk of hiatus hernia recurrence and ongoing reflux symptoms, and the potential need to add a bariatric procedure at the time of hiatus hernia repair or at a later date. [5–11]

The current obesity literature is limited to surgery for gastroesophageal reflux disease with or without a small hiatus hernia. These studies demonstrate that clinical outcomes following antireflux surgery in obese individuals are excellent, and in general, comparable to non-obese patients. [12–16] However, the risk of recurrent reflux symptoms appears to be greater in obese patients. [1, 17, 18]

Surgery for large hiatus hernia is more complex than for reflux in patients with a small hernia. It requires greater thoracic dissection to reduce the hernia contents back into the abdomen, and the greater the percentage of intrathoracic stomach, the higher the risk of postoperative morbidity and ongoing reflux. [19, 20] Currently, there is minimal data available to inform the outcomes for large hiatus hernia repair in obese and morbidly obese patients. Furthermore, the literature is confused by the lack of a universally accepted definition for a “large” hiatus hernia. Definitions include hernia containing more than 30% or 50% of the stomach, hernias measuring more than  ≥ 7 cm in length, crural defects ≥ 5 cm in diameter, and crural defects surface area of ≥ 10 cm.^2^. [21–27]

In this study, we assessed a large cohort of patients who underwent elective repair of large hiatus hernias defined as containing at least 50% of the stomach to determine and compare safety and early clinical outcomes in non-obese, obese, and morbidly obese patient groups. We hypothesised that large hiatus hernia repair in obese and morbidly obese patients would be associated with a higher rate of perioperative morbidity and early hernia and symptom recurrence compared to non-obese patients.

## Materials and methods

### Data source and participants characteristics

A retrospective analysis was conducted of data from a prospective database which contained perioperative and outcome data for patients who underwent surgery for a large hiatus hernia between January 2000 and December 2023 at Flinders Medical Centre, Royal Adelaide Hospital and associated private hospitals in Adelaide, South Australia. Patients were included in this study if they had an elective operation for a large hiatus hernia identified by preoperative endoscopy, oral contrast X-ray study or CT, and subsequently confirmed at the time of surgery. Large hiatus hernia was defined as a hernia containing at least 50% of the stomach. This definition was chosen as it has been used elsewhere, it is conservative and does not include any patients with hernias that would be considered less than large in any classification system, and size can be confirmed on cross-sectional imaging and at laparoscopy. [19] Only patients for whom their BMI at surgery was known were included. Patients were excluded if BMI data were not available, they were underweight (BMI < 18.5 kg/m^2^), underwent emergency or revisional surgery, or had concomitant bariatric surgery in addition to the hiatus hernia repair. Patients who had surgery before January 2000 were also excluded to ensure any learning curve bias for laparoscopic repair was avoided as by then all consultant surgeons had then performed at least 40 previous operations. [28] All operations were either performed or assisted by a consultant upper gastrointestinal surgeon.

Patients underwent preoperative endoscopy to assess for esophagitis, Barrett’s oesophagus, Cameron’s ulcers, and hernia size and to exclude underlying malignancy. Contrast swallow radiology (CT or X-ray) was usually performed to assess hernia size and type. Esophageal manometry and 24-h pH studies were performed at the surgeon’s discretion when patients were being considered for surgery for reflux. When undergoing surgery for mechanical problems, but not reflux, an anterior partial fundoplication was routinely performed and these tests were usually not required. Prior to surgery, patients also underwent routine blood tests within 2 weeks prior to surgery and any clinically significant anaemia was corrected with iron infusion.

### Surgical procedure

A standardised surgical approach was performed across all sites and has been described previously. [29] The steps included complete hiatal sac dissection, reduction of the entire sac, stomach, and lower 2–3 cm of distal oesophagus back into the abdominal cavity, tension-free sutured repair of the widened esophageal hiatus with posterior and supplemental anterior stitches as required, and a fundoplication to anchor the stomach below the diaphragm and control reflux. The type of fundoplication was tailored to the clinical presentation. A Nissen or anterior 180 degree partial fundoplication was constructed when reflux control was a significant component of the presentation, whereas an anterior 90^0^ partial wrap was often used as a gastropexy to minimise the risk of post-fundoplication side effects when patients presented with mechanical issues from the large hiatus hernia, but not reflux. Hiatal mesh reinforcement was rarely performed. The majority of cases where mesh reinforcement was performed were part of a randomised controlled trial comparing sutured versus absorbable versus non-absorbable mesh repair or in the lead in phase to that trial. [30] Beyond the trial, mesh was seldom used.

A contrast swallow was routinely performed on the day after surgery to ensure repair integrity and, if there were any radiological concerns, early laparoscopic re-exploration was undertaken. Later investigations were only performed in symptomatic patients. An early hernia recurrence was defined as occurring during the patient’s admission for the initial operation, and usually identified in the context of a routine day one postoperative contrast swallow X-ray study. A late hernia recurrence and reoperation was defined as occurring after discharge, but within 24 months following the original surgery. Hernia recurrences were classified as small (≤ 2 cm), medium (> 2 cm but < 5 cm), or large (≥5 cm) based on contrast swallow X-rays or endoscopy assessment.

### Perioperative outcomes

Patients were categorised into 3 groups based on BMI: non-obese (BMI < 30 kg/m^2^), obese (BMI 30–34.9 kg/m^2^), and morbidly obese (BMI ≥ 35 kg/m^2^). Perioperative outcomes that were measured include length of stay, all complication, major complication (defined as Clavien–Dindo ≥ 3a), early reoperation, and 30-day and 90-day mortality rates. Late symptomatic hernia recurrence and reoperations were also determined.

### Patient follow-up and symptom outcomes

Patients prospectively completed a structured questionnaire preoperatively, at 3, 6, and 12 months postoperatively and annually thereafter. The questionnaire included a yes/no section regarding the presence or absence of various symptoms: Heartburn, regurgitation, dysphagia, nausea and vomiting, shortness of breath, and nocturnal cough. Heartburn, regurgitation, dysphagia severity scores, and overall satisfaction scores were also determined (measured on a visual analogue scales 0–10). A yes/no question was also asked to determine if patients believed their original decision to undergo surgery was correct. We compared preoperative and 12-month postoperative time points. To maximise follow-up, if 12-month data were not available, but 2-year data were available, then this was substituted. If 2-year data were also missing, then 6-month followed by 3-month data were substituted in that order.

### Statistical analysis

Categorical data were analysed using Fisher’s exact or Pearson’s chi-square test. Continuous variables were compared using ANOVA or independent sample *t* tests. Multivariable-adjusted models were then performed to adjust for potential confounders of age and sex to determine the association of outcomes with BMI. Generalised linear models with gamma distribution were employed to determine differences for length of stay; logistic regression models were applied to determine the risk for complications, mortality, hernia recurrence, and reoperation rates. Any missing data points were not included in the analysis.

A *p* value < 0.05 was considered significant for all baseline variables and multivariable regression analyses. Statistical analysis was performed using IBMs Statistical Package for the Social Sciences (SPSS; version 19 for Apple Macintosh).

This study has been reported in accordance with the Strengthening the Reporting of Observational Studies in Epidemiology (STROBE) guidelines to ensure transparency and completeness in the reporting of observational research. Ethics approval was provided by the Southern Adelaide Clinical Research Ethics Committee (approval numbers: 145.23, 110.16, 12.14, 10.056).

## Results

### Patient demographics

**Fig. 1:**
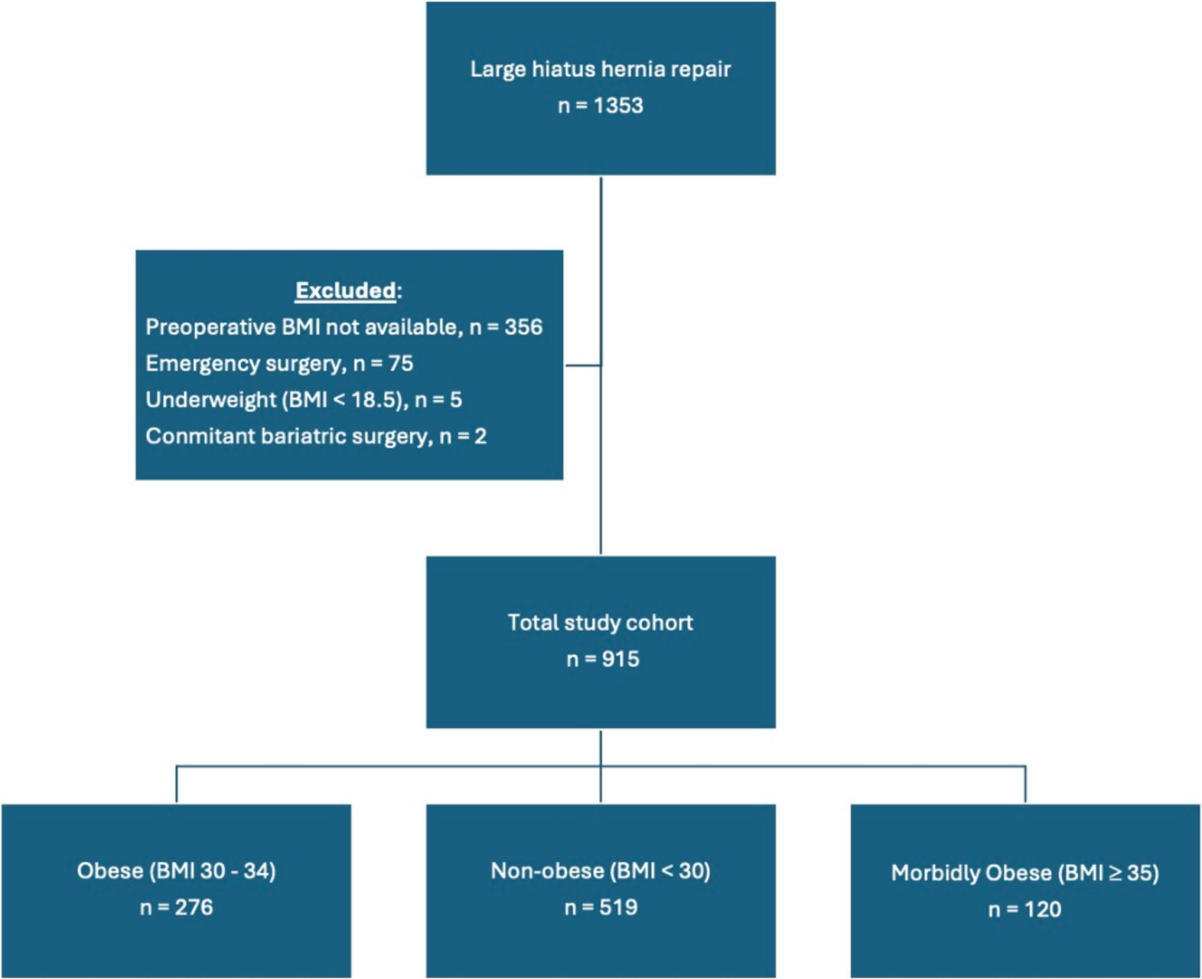
Patients included in study

1353 patients who underwent repair of a large hiatus hernia were identified. 997 patients had their preoperative BMI recorded. Seventy-five of these patients were excluded as they had undergone emergency or urgent surgery for gastric volvulus or an obstructing hiatus hernia. Five patients were underweight (BMI ≤ 18.5 kg/m^2^) and excluded. Two patients had concomitant bariatric surgery in addition to hiatus hernia surgery and were also excluded. In total, 915 patients were included for analysis (Fig. 1). The mean age of the cohort at surgery was 67.5 (SD, 10.4) years, with BMI of 29.7 (SD, 4.8) kg/m^2^, and 640 (69.9%) were female. 519 (56.7%) patients had a BMI < 30 kg/m^2^, 276 (30.1%) had BMI between 30 and 34 kg/m^2^, and 120 (13.1%) had BMI ≥ 35 kg/m^2^. Morbidly obese and obese patients were more likely to be younger and female compared to non-obese patients. Morbidly obese patients were also more likely to have pre-existing diabetes (Table 1).

**Table 1:** Baseline characteristics of patients undergoing repair of a large hiatus hernia

	BMI < 30 *n* = *519*	BMI 30–34 *n* = *276*	BMI ≥ 35 *n* = *120*	*p* value
Demographics				
Age, years	69.19 (68.29–70.09)	65.76 (64.58–66.95)	64.00 (62.22–65.78)	< 0.001
Female (n%)	316 (60.9%)	220 (79.7%)	104 (83.9%)	< 0.001
Height (m)	1.67 (1.65–1.67)	1.62 (1.61–1.63)	1.61 (1.60–1.63)	< 0.001
Weight (kg)	73.47 (72.56–74.39)	84.70 (83.34–96.05)	100.03 (97.63–102.42)	< 0.001
Body mass index, kg/m^2^	26.37 (26.18–26.58)	32.07 (31.90–32.23)	38.31 (37.72–38.92)	< 0.001
Comorbidity (n%)				
Cardiovascular	96 / 497 (19.3%)	37 / 261 (14.2%)	18 / 109 (16.5%)	0.201
Respiratory	136 / 499 (27.3%)	83 / 261 (31.8%)	41 / 114 (36.0%)	0.128
Diabetes	30 /490 (6.1%)	16 / 276 (5.8%)	17 / 106 (16.0%)	< 0.001
Previous abdominal surgery	295 / 501 (58.9%)	157 / 276 (56.9%)	71 / 114 (62.3%)	0.611

Data are mean (standard deviation / 95% confidence intervals) or number (%)

### Surgical demographics and outcomes

Reasons for surgery are outlined in Table 2. Heartburn was the most common reason for surgery across all cohorts. Bleeding, anaemia, and shortness of breath were commonest in the morbidly obese group, whereas epigastric pain, nausea, and vomiting were more common in the non-obese group. Most patients underwent preoperative endoscopy. Ulcerative esophagitis (LA grade B–D) was seen more frequently in the non-obese group.

**Table 2:** Preoperative details for all patients undergoing repair of a large hiatus hernia

	BMI < 30 *n* = *288*	BMI 30–35 *n* = *170*	BMI ≥ 35 *n* = *84*	*p*-value
Reason(s) for Surgery (n%)				
Heartburn	143 (49.7%)	94 (55.3%)	45 (53.6%)	0.482
Regurgitation	103 (35.8%)	62 (36.5%)	25 (29.8%)	0.536
Bleeding / Anaemia	71 (24.7%)	51 (30.0%)	35 (41.7%)	0.010
Epigastric Pain	16 (5.6%)	2 (1.2%)	2 (2.4%)	0.044
Dysphagia	21 (7.3%)	15 (8.8%)	10 (11.9%)	0.403
Nausea and Vomiting	12 (4.2%)	0 (0%)	1 (1.2%)	0.014
Shortness of Breath	12 (4.2%)	6 (3.5%)	10 (11.9%)	0.010
Cough	12 (4.9%)	8 (4.7%)	2 (2.4%)	0.609
Duration of Sex (months)	7.28 (6.49–8.08)	7.76 (6.74–8.78)	7.31 (5.92–8.70)	0.757
Preoperative Investigations				
Endoscopy	485 / 519 (93.4%)	258 / 276 (93.5%)	114 / 120 (95.0%)	0.812
Esophagitis	147 / 485 (30.3%)	74 / 258 (28.7%)	21 / 113 (18.6%)	0.044
Barrett’s oesophagus	54 / 493 (11.0%)	26 / 262 (9.9%)	9 / 116 (7.8%)	0.583
Cameron’s Ulcers	21 / 485 (4.3%)	10 / 258 (3.9%)	6 / 114 (5.3%)	0.832

Patients may have multiple reasons for surgery

Data are mean (standard deviation / 95% confidence intervals), or number (%)

Operative findings are summarised in Table 3. Hernia size and type did not differ. The majority of patients (98.8%) underwent a laparoscopic procedure and there were no differences in conversion rates. There were no differences in hiatal suture number, position, or the use of mesh. Morbidly obese patients were more likely to have a posterior 270° or total 360° fundoplication constructed. Only 1 (0.11%) patient underwent a Collis-gastroplasty, and no one had a gastrostomy tube placed. One (0.11%) patient did not have a fundoplication performed. There were no significant differences in duration of surgery or intraoperative complications.

**Table 3:** Operative details for all patients undergoing repair of a large hiatus hernia

	BMI < 30 *n* = *519*	BMI 30–34 *n* = *276*	BMI ≥ 35 *n* = *120*	*p* value
Hernia Size				
> 50% intrathoracic	416 / 519 (80.2%)	238 / 276 (86.2%)	101 / 120 (84.2%)	0.087
Total intrathoracic	103 / 519 (19.8%)	38 / 276 (13.8%)	19 / 120 (15.8%)	
Hernia Type				
Sliding	137 / 504 (27.2%)	70 / 270 (25.9%)	30 / 116 (25.9%)	0.540
Rolling / Paraesophageal	95 / 504 (18.8%)	44 / 270 (16.3%)	27 / 116 (23.3%)	
Mixed	272 / 504 (54.0%)	156 / 270 (57.8%)	59 / 116 (50.9%)	
Approach				
Laparoscopic	480 / 486 (98.8%)	274 / 276 (99.3%)	117 / 120 (97.5%)	0.592
Conversion: Lap to Open	4 / 486 (0.8%)	2 / 276 (0.7%)	2 / 120 (1.7%)	
Open	2 / 486 (0.4%)	0 / 276 (0%)	1 / 120 (0.8%)	
Reason for Conversion				
Unable to reduce hernia sac	3 / 4 (75%)	1 / 2 (50%)	0 / 2 (0%)	0.314
Adhesions	0 / 4 (0%)	0 / 2 (0%)	1 / 2 (50%)	
Bleeding	0 / 4 (0%)	1 / 2 (50%)	1 / 2 (50%)	
Esophageal Perforation	1 / 4 (25%)	0 / 2 (0%)	0 / 2(0%)	
Hiatal Repair				
Number of sutures	4.92 (4.89–5.05)	4.77 (4.59–4.95)	4.6 (4.31–4.89)	0.109
Suture placement				
Anterior	5 / 507 (1.0%)	0 / 270 (0%)	0 / 118 (0%)	0.158
Anterior + Posterior	205 / 507 (40.4%)	98 / 270 (36.3%)	40 / 118 (33.9%)	
Posterior	297 / 507 (58.6%)	172 / 270 (63.7%)	78 / 118 (66.1%)	
Mesh				
Mesh Used	34 / 519 (6.6%)	15 / 276 (5.4%)	7 / 120 (5.8%)	0.814
Mesh Type				
Absorbable	18 / 34 (52.9%)	12 / 15 (80.0%)	5 / 7 (71.4%)	0.172
Non-absorbable	16 / 34 (47.1%)	3 / 15 (20.0%)	2 / 7 (28.6%)	
Fundoplication				
Wrap performed	516 / 516 (100%)	275 / 275 (100%)	119 / 120 (99.2%)	
Fundoplication Type				
90 Anterior	62 / 516 (12.0%)	30 / 275 (10.9%)	20 / 119 (16.8%)	0.050
180 Anterior	387 / 516 (75.0%)	216 / 275 (78.5%)	76 / 119 (63.9%)	
270 Posterior	27 / 516 (5.2%)	17 / 275 (6.2%)	12 / 119 (10.1%)	
360 Nissen	40 / 516 (7.8%)	12 / 275 (4.4%)	11 / 119 (10.1%)	
Short Gastric Divided	37 / 515 (7.2%)	17 / 273 (6.2%)	14 / 119 (11.8%)	0.147
Bougie Used	252 / 514 (49.0%)	134 / 271 (49.4%)	39 / 119 (32.8%)	0.004
Drain Used	23 / 398 (5.8%)	8 / 223 (3.6%)	2 / 88 (2.3%)	0.243
Gastrostomy	0 (0%)	0 (0%)	0 (0%)	
Collis-gastroplasty	1 / 483 (0.2%)	0 / 264 (0%)	0 / 111 (0%)	0.678
Intraoperative Complication	20 / 487 (4.1%)	12 / 267 (4.5%)	6 / 112 (5.4%)	0.840
Duration of Surgery (min)	106.44 (102.70–110.18)	103.41 (98.94–107.88)	113.61 (105.91–121.31)	0.074

Postoperative complications are detailed in Table 4. Major complications (Clavien–Dindo ≥ 3a) and return to the operating room rates did not differ. There were only 2 mortalities (0.22%) at 90 days. A symptomatic hernia recurrence was detected by endoscopy or barium swallow within 24 months of surgery in 33 (3.6%) patients. The majority (66.7%) of these were small in size (< 2 cm). 12 (1.3%) patients underwent reoperation. There were no differences in hernia recurrence or reoperation rates between the 3 groups.

**Table 4:** Postoperative outcomes for all patients undergoing repair of a large hiatus hernia

	BMI < 30 *n* = *519*	BMI 30–34 *n* = *276*	BMI ≥ 35 *n* = *120*	*p* value
Length of Stay (Days)	2.80 (2.61–3.00)	2.48 (2.32–2.64)	2.57 (2.28–2.85)	0.063
Postoperative complications				
All complications	56 / 506 (11.1%)	17 / 274 (6.2%)	9 / 117 (7.7%)	0.067
Major (Clavien–Dindo ≥ 3)	20 / 505 (4.0%)	8 / 272 (2.9%)	2 / 116 (1.7%)	0.435
Complication Details				
Grade 3	18 / 20 (90%)	5 / 8 (62.5%)	2 / 2 (100%)	
Grade 4	2 / 20 (10%)	2 / 8 (25.0%)	0 / 2 (0%)	0.332
Grade 5	0 / 20 (0%)	1 / 8 (12.5%)	0 / 2 (0%)	
Return to theatre (RTT)				
RTT required	14 / 519 (2.7%)	4 / 276 (1.14)	2 / 120 (1.7%)	0.475
Reason				
Early Recurrence	9 / 14 (64.3%)	2 / 4 (50.0%)	2 / 2 (100%)	0.442
Dysphagia	3 / 14 (21.4%)	1 / 4 (25.0%)	0 / 2 (0%)	
Esophageal leak	2 / 14 (14.3%)	0 / 4 (0%)	0 / 2 (0%)	
Sepsis	0 / 14 (0%)	1 / 4 (25.0%)	0 / 2 (0%)	
Death				
30-day mortality	0 / 519 (0%)	1 / 276 (0.4%)	0 / 120 (0%)	
90-day mortality	1 /519 (0.2%)	1 / 276 (0.4%)	0 / 120 (0%)	
Late Recurrence (within 24 m)				
Symptomatic recurrence	17 / 519 (3.3%)	11 / 276 (4.0%)	5 / 120 (4.2%)	0.827
Recurrence Size				
Small (≤ 2 cm)	11 / 17 (64.7%)	7 / 11 (63.6%)	4 / 5 (80.0%)	0.935
Medium (> 2 and < 5 cm)	4 / 17 (23.5%)	3 / 11 (27.3%)	1 / 5 (20.0%)	
Large (≥ 5 cm)	2 / 17 (11.8%)	1 / 11 (9.1%)	0 / 5 (0%)	
Reoperation for late recurrence (≤ 24 months)	5 / 519 (1.0%)	5 / 276 (1.8%)	2 / 120 (1.7%)	0.569

Data are mean (standard deviation / 95% confidence intervals) or number (%)

Heartburn, regurgitation, and dysphagia severity scores and clinical satisfaction scores are summarised in Table 5. At median 12-month follow-up (mean 11.2 months), heartburn, regurgitation, and dysphagia (solid and liquid) severity scores were significantly lower in all groups (*p* < 0.001 for all pre- and postoperative comparisons). Non-obese patients had lower heartburn severity scores than obese and morbidly obese patients. Overall satisfaction was high in all groups, and the vast majority of patients (97.4%) believed they made the correct decision to undergo surgery.

**Table 5:** Symptom and satisfaction scores for all patients undergoing repair of a large hiatus hernia

	BMI < 30	BMI 30–34	BMI ≥ 35	*p* value
Heartburn (VAS 0–10)				
Preoperative Score	7.32 (6.90–7.74)	8.08 (7.55–8.62)	7.05 (6.00–8.11)	0.067
Postoperative Score	0.94 (0.69–1.18)	1.86 (1.36–2.36)	1.21 (0.63–1.78)	0.010
Regurgitation (VAS 0–10)				
Preoperative Score	6.89 (6.33–7.44)	7.35 (6.62–8.09)	6.68 (5.48–7.88)	0.509
Postoperative Score	1.02 (0.69–1.36)	1.30 (0.81–1.79)	1.74 (0.91–2.57)	0.185
Dysphagia Solids (VAS 0–10)				
Preoperative Score	5.57 (5.05–6.09)	6.29 (5.59–7.00)	6.25 (5.22–7.28)	0.193
Postoperative Score	1.23 (0.95–1.51)	1.55 (1.12–1.97)	1.61 (0.96–2.25)	0.321
Dysphagia Liquids (VAS 0–10)				
Preoperative Score	4.66 (4.05–5.28)	5.13 (4.26–5.99)	5.73 (4.44–7.02)	0.269
Postoperative Score	0.57 (0.39–0.75)	0.67 (0.35–0.98)	0.76 (0.31–1.23)	0.658
Satisfaction Score (out of 10)	8.74 (8.50–8.98)	8.62 (8.18–9.06)	8.91 (8.33–9.48)	0.702
Would repeat surg (yes/no), %	229 / 243 (94.2%)	115 / 121 (95.0%)	51 / 53 (96.2%)	0.655

Data are mean (standard deviation / 95% confidence intervals) or number (%)

### Multivariable analysis for risk factors of surgical outcomes

As older age and male sex were independently associated with increased surgical morbidity after hiatus hernia repair, a multivariable regression analysis was performed. The effect of BMI was assessed categorically on postoperative outcomes after adjusting for age and sex. There were no statistically significant differences in length of stay, intraoperative and postoperative complications, 90-day mortality, hiatus hernia recurrence, and reoperation rates when comparing non-obese vs obese vs morbidly obese patients. (Table 6).

**Table 6:** Association of BMI with surgical outcomes after adjusting for age and sex

	Length of Stay		Intraoperative Complications		Postoperative Complications	
	OR	*p* value	OR	*p* value	OR	*p* value
Non-obese	1 [Reference]		1 [Reference]		1 [Reference]	
Obese	− 0.16 (− 0.43, 0.12)	0.259	1.22 (0.55, 2.6)	0.600	0.69 (0.37, 1.24)	0.234
Morbidly obese	− 0.03 (− 0.4, 0.35)	0.885	1.44 (0.49, 3.67)	0.465	0.89 (0.38, 1.84)	0.767

	90-day Mortality		Recurrent Hernia < 24 months		Reoperation for recurrent hernia	
	OR	*p* value	OR	*p* value	OR	*p* value
Non-obese	1 [Reference]		1 [Reference]		1 [Reference]	
Obese	0.95 (0.51, 1.71)	0.878	0.88 (0.46, 1.63)	0.705	0.91 (0.39, 2.02)	0.828
Morbidly obese	0.65 (0.22, 1.61)	0.398	1.14 (0.51, 2.40)	0.723	1.15 (0.39, 2.95)	0.778

## Discussion

Contrary to our hypothesis, our study demonstrated that large hiatus hernia repair in obese and morbidly obese patients is a safe and effective treatment option, with comparable outcomes to non-obese patients. We found no differences in operative time, laparoscopic conversion to open rates, or length of stay. All adverse events (intraoperative and postoperative complications, early return to theatre, and mortality rates) were acceptably low and similar across all groups. Despite some literature suggesting that a symptomatic recurrent hiatus hernia is significantly greater in obese patients, we found no such differences. [9] Furthermore, all groups experienced a significant improvement in heartburn, regurgitation, and dysphagia severity, as well as high overall satisfaction scores.

We found that obese and morbidly obese patients were more likely to be younger and female. Han et al., also reported both these findings in their study. [31] This is likely due to selection bias, as surgeons are less inclined to operate on patients who are both elderly and obese. The discrepancy in gender is an interesting finding and possibly relates to different fat distribution patterns. Obese males are more likely to have increased visceral fat. [32–34] Increased visceral adiposity has been shown to increase operating time, blood loss, and perioperative morbidity during laparoscopic surgery. [35, 36] Furthermore, excessive visceral fat is strongly associated with significant comorbidities including insulin resistance and coronary artery disease. [37, 38] As such, surgeons may be less inclined to operate on obese males than females due to both technical- and health-related concerns, although the surgeons contributing to this study did not consciously avoid operating on men.

Our study found that whilst heartburn was the most common reason for surgery, there were no significant differences between obese and non-obese patients. Interestingly, anaemia was a frequent preoperative finding, and this was highest in the morbidly obese group (41.7%). Carrott et al. also found that preoperative anaemia is a common finding in patients with a large hiatus hernia (45.6%) that resolved in 71% of patients following surgical repair. [39] There is also a strong association between obesity and anaemia, which likely reflects the higher rate seen in this demographic. [40] Despite similar rates of heartburn, obese patients were less likely to demonstrate macroscopic esophagitis. Kim et al. suggest that central obesity is also associated with non-erosive esophagitis. [41]

Prior to our study, Han et al. published the largest study to assess the impact of BMI on large hiatal hernia repair. Of the 884 patients included, 725 (86.3%) had an intrathoracic stomach of 50% or more and 45 patients (5.1%) underwent an emergency procedure. They demonstrated similar findings that increasing BMI was not associated with increased perioperative blood loss, length of stay, major complications, 90-day mortality, or early recurrence. However, their study had several limitations. The cohort was split across six categories based on BMI (underweight, normal weight, pre-obesity, and obesity class 1, 2, and 3) for comparison. Obesity class II had 76 (8.6%) patients and class III had just 27 (3.1%) patients. These groups may be underpowered to detect any significant differences. Furthermore only 24.8% of patients included in their study underwent a standard minimally invasive approach, and this reduced to 7.8% for the obesity class 3 group. Finally, symptom outcomes were not measured to determine treatment success. [31]

Our study expands on previous work by reporting a larger cohort of patients. We used stricter criteria by only including patients with a large hiatus hernia (≥50% intrathoracic stomach). We also excluded patients that underwent emergency surgery or were underweight (BMI ≤ 18.5 kg/m.^2^) as they have been shown to independently increase perioperative morbidity and mortality. [42]

However, our study does have several limitations. It is retrospective analysis of a prospectively collected audit and outcome data, and it is non-randomised. Baseline demographics were not homogeneous as obese and morbidly obese patients were more likely to be younger, female, and have diabetes. This is significant as being older and male sex can be associated with poorer surgical outcomes. [42] As such, a multivariable regression analysis was performed to minimise the effect of these confounders. There were missing datapoints for some patients. However, this is somewhat mitigated by the large sample size. Also, follow-up investigations were limited to symptomatic patients and were performed at surgeon discretion. Thus, asymptomatic recurrences are not all identified, and the true number of recurrent hiatus hernias is likely not captured. However, the clinically important symptomatic hernias and those requiring further surgery were all identified.

The role of bariatric surgery with large hiatus hernia repair is controversial. Traditionally, sleeve gastrectomy has been avoided due to concerns it would worsen gastroesophageal reflux. A systematic review of simultaneous sleeve gastrectomy with hiatus hernia review in obese patients found that it was safe and generally effective, but prevalence of post-operative gastroesophageal reflux was 29.7%. [43] The majority of the 18 included studies were small volume case series that were retrospective in nature. Hiatus hernia size is also either small to moderate in size or not mentioned at all. Roux-en-Y gastric bypass is considered the gold standard for morbidly obese patients with significant reflux symptoms. [44] However, its role in managing a large hiatus hernia is largely unknown. Kollman et al. identified 12 patients over a 10-year period who underwent simultaneous large hiatal hernia (> 5 cm) repair with Roux-en-Y gastric bypass. They found that whilst operating time was longer compared to Roux-en-Y gastric bypass alone, major complications, reoperations, and length of stay did not differ. [45] Other studies have demonstrated similar safety outcomes. [46, 47] DuCoin et al. compared large hiatus hernia repair with and without gastric bypass. Of the 16 / 40 patients that underwent antireflux gastric bypass, they found no statistical differences in reflux resolution or hiatus hernia recurrence. [11] However, surgeons may be apprehensive to combine large hiatus repair with gastric bypass for several reasons. These include longer operative time, increased surgical risks (e.g. stomal ulcers, anastomotic leak, and internal hernia), and a risk of intrathoracic pouch migration which may worsen reflux symptoms in the longer term. [48]

Surgeons may still recommend short-term preoperative weight loss prior to large hiatus hernia repair to overcome some of the technical challenges encountered by operating on obese patients. As central adiposity increases, port access, positioning, and excessive torque may occur. [49, 50] Furthermore, increased intra-abdominal fat and an enlarged steatotic left liver lobe can obscure the hiatus. [51]

## Conclusion

Despite the technical- and health-related challenges, our study has demonstrated that large hiatus hernia repair in obese and morbidly obese patients is safe and effective. Operative metrics, complications, and recurrence rates were similar across BMI groups, and all patients experienced significant symptom relief and high satisfaction. Surgery should not be withheld based on BMI alone.
